# Phase Equilibria of the V-Ti-Fe System and Its Applications in the Design of Novel Hydrogen Permeable Alloys

**DOI:** 10.3390/membranes13100813

**Published:** 2023-09-27

**Authors:** Yihao Wang, Limin Jia, Erhu Yan, Zhijie Guo, Shuo Zhang, Tangwei Li, Yongjin Zou, Hailiang Chu, Huanzhi Zhang, Fen Xu, Lixian Sun

**Affiliations:** 1 Guangxi Key Laboratory of Information Materials, Guilin University of Electronic Technology, Guilin 541004, China; wabgyihao0034@163.com (Y.W.); guozhijie000002@163.com (Z.G.); zhangshuo_0902@163.com (S.Z.); litangwei_1222@163.com (T.L.); zouy@guet.edu.cn (Y.Z.); chuhailiang@guet.edu.cn (H.C.); zhanghuanzhi@guet.edu.cn (H.Z.); xufen@guet.edu.cn (F.X.); sunlx@guet.edu.cn (L.S.); 2 Hebei Key Laboratory of Material Near-Net Forming Technology, School of Materials Science and Engineering, Hebei University of Science and Technology, Shijiazhuang 050018, China

**Keywords:** V-Ti-Fe alloy, phase equilibria, hydrogen permeability

## Abstract

The precise liquidus projection of the V-Ti-Fe system are crucial for designing high-performance hydrogen permeation alloys, but there are still many controversies in the research of this system. To this end, this article first uses the CALPHAD (CALculation of PHAse Diagrams) method to reconstruct the alloy phase diagram and compares and analyses existing experimental data, confirming that the newly constructed phase diagram in this article has good reliability and accuracy. Second, this obtained phase diagram was applied to the subsequent development process of hydrogen permeation alloys, and the (Ti_65_Fe_35_)_100−x_V_x_ (x = 0, 2.5, 5, 10, 15, 25) alloys with dual-phase {bcc-(V, Ti) + TiFe} structure were successfully explored. In particular, the alloys with x values equal to 2.5 at.% and 5 at.% exhibit relatively high hydrogen permeability. Third, to further increase the H_2_ flux permeation through the alloys, a 500-mm-long tubular (Ti_65_Fe_35_)_95_V_5_ membrane for hydrogen permeation was prepared for the first time. Hydrogen permeation testing showed that this membrane had a very high H_2_ flux (4.06 mL min^−1^), which is ca. 6.7 times greater than the plate-like counterpart (0.61 mL min^−1^) under the same test conditions. This work not only indicates the reliability of the obtained V-Ti-Fe phase diagram in developing new hydrogen permeation alloys, but also demonstrates that preparing tubular membranes is one of the most important means of improving H_2_ flux.

## 1. Introduction

**Figure 1:**
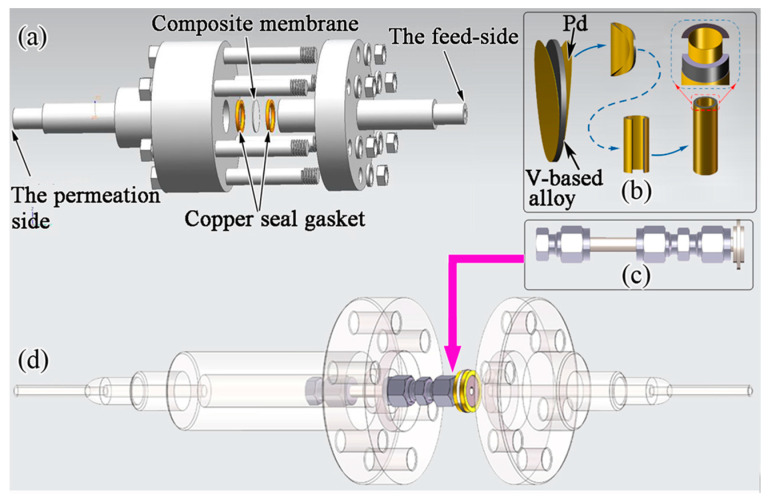
Schematic of the hydrogen permeation membrane module for sheet (**a**) and tubular (**d**) membrane. (**b**,**c**) represent the process of transition from the sandwiched sheet composite membrane to tubular membrane and the final structure of tubular membrane, respectively.

Recently, group 5B metals (Nb, V, Ta) and homologous alloys have been considered potential candidates to replace the benchmark Pd-based hydrogen permeation membrane [1,2,3]. In particular, V- or Nb-based multi-phase alloys (e.g., Nb-TiCo [4,5], Nb-TiNi [6,7,8], V-TiNi [9], V-Al [10]) have been widely studied due to their low-price advantage, favourable hydride phase stability, and excellent mechanical properties. To increase the catalytic activity to H_2_, a dense Pd layer of ~200 nm is usually deposited on the alloy surface, and the schematic diagram of these composite membranes is shown in Figure 1a,b. In 2013, Ishikawa et al. [11] demonstrated that Nb-Ti-Fe alloys, which are composed of the bcc-(Nb, Ti) and TiFe phases, show higher hydrogen permeation performance than that of the Nb-TiNi/TiCo alloys. Shortly afterwards, Nb_5_Ti_60_Fe_35_, with the highest permeability in this system, was developed by the present authors [12]. As is well known, V and Nb, both belonging to the 5B group, have similar chemical and physical properties as well as electronic structures [6,7,8,9,13,14]. Moreover, V has the ability to maintain bcc structure over a wider composition range than Ta or Nb [15]. If Nb in the Nb-Ti-Fe alloy is replaced by V to form a new alloy system, i.e., V-Ti-Fe system, it is strongly expected to obtain promising dual-phase materials that can be used in hydrogen purification.

**Figure 2:**
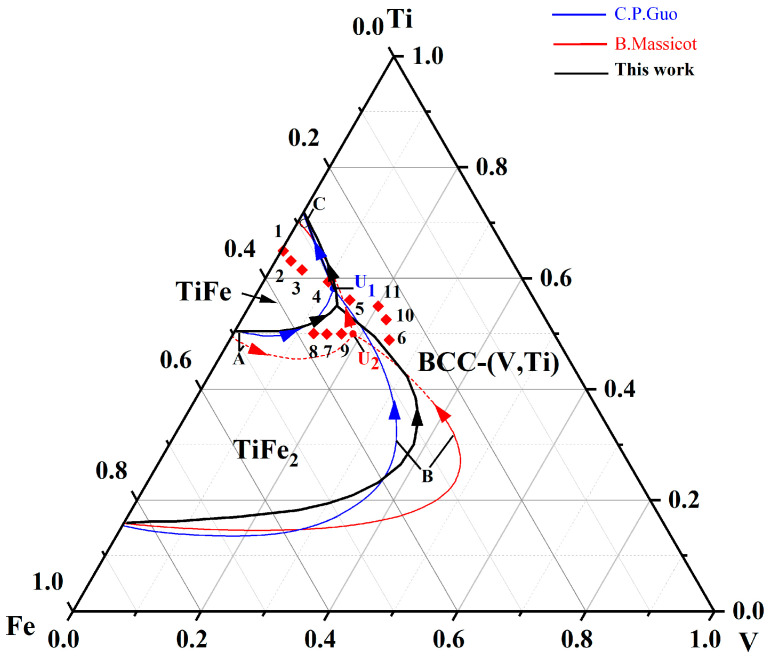
Calculated phase diagram for the V-TiFe system and the position of the studied alloys (#1–#11) on the liquidus projection (solid blue line) of this system. The black dashed line and pink dotted line represent the results reported by Guo et al. [16] and Massicot et al. [17], respectively. The red boxes represent the composition of the studied alloys.

However, constrained by the precise equilibrium phase diagram of the V-Ti-Fe system, no hydrogen permeation alloy has been explored so far. Although Guo et al. [16] and Massicot et al. [17] have successively reported on the isothermal sections and phase equilibria of this system, there are still many contradictions and controversies, especially in key parameters such as the composition of ternary invariant transformations (the results for Massicot and Guo are U′ and U″, respectively) and the range size of phase zone (TiFe, TiFe_2_ and bcc-(V, Ti)), as shown in Figure 2. The contradictory parameters in this phase diagram make the alloy design extremely difficult. Therefore, it is necessary to reconstruct a more accurate phase diagram to clarify the above contradictions and use it to guide the development of hydrogen permeation alloys in this system.

In addition, the hydrogen permeation performance of the membranes mentioned above is commonly measured using gas permeation technology, and most of them are sheet-like membranes, as shown in Figure 1a. The advantage of the thin film being in a “flake-like” shape is that it not only requires fewer raw materials, but also facilitates subsequent processing [18]. However, this structure will limit their effective hydrogen permeation area (only 113 mm^2^), resulting in low hydrogen permeation flux that cannot meet practical needs [19]. Expanding the sheet-like membrane area will increase the risk of alloy membrane rupture. If a tubular membrane is prepared, it may solve the above problem. On the one hand, extending the length of the tube can increase the effective hydrogen permeation area and the total H_2_ flux, but on the other hand, controlling the diameter of the tube can avoid membrane rupture, as shown in Figure 1b–d. However, so far, what the literature has not reported is whether it is possible to achieve a significant increase in total H_2_ flux by preparing tubular membranes. In theory, increasing the length of the tubular film can avoid problems such as low hydrogen permeation flux caused by the alloy having a lower hydrogen permeability.

With these considerations in mind, the present work is dedicated to conducting research on the thermodynamic calculation of the V-Ti-Fe phase diagram and obtaining more accurate phase equilibrium data by coupling it with experimental determination. Based on this, a new alloy series with the general formula (Ti_65_Fe_35_)_100−x_V_x_, where x varies from 0 to 25 at.%, was designed, and their microstructural properties and hydrogen permeability were investigated systematically. Finally, this article explores the possibility of preparing tubular hydrogen permeation membranes and applying them to the hydrogen permeation process within the selected testing temperature range.

## 2. Experimental and Thermodynamic Model

### 2.1. Experimental Detail

The V-Ti-Fe ingots were prepared by using induction skull melting (ISM) under a high-purity (5N) argon atmosphere, and the purity of these raw materials (Beijing Dream Material Technology Fe., Ltd., Beijing, China) was 99.99 at.%. for all. For convenience of discussion, the alloy compositions are numbered #1–#11, as shown in Table 1. After cutting by electric discharge wire-cutting, disk (φ16 mm × 0.7 mm) and tubular (φ6 mm × 60 mm) samples were prepared and polished as the substrate for chemical Pd plating. After sensitisation-activation, Pd particles were deposited on the surface of the substrate through the chemical plating process, which was carried out in a magnetic stirring apparatus. The components and experimental conditions are listed in Table 2. After plating, each sample was washed five times with deionised water and dried in a vacuum drying oven at 100 °C for 3 h. 

Next, all samples were placed in the hydrogen permeation test module to measure their hydrogen permeation performance by using conventional gas-permeation techniques. The testing temperature and pressure were 523–673 K and 0.1–0.5 MPa, respectively. Detailed steps for hydrogen permeation measurement are described in our previous work [20,21]. To obtain the hydrogen solubility, the hydrogen absorption test was also conducted for all the samples using a standard Sieverts-type device. After activation by four absorbing-desorbing cycles, pressure-composition isotherms were measured and recorded at 673 K and up to a hydrogen pressure of 0.6 MPa. 

The crystal structures of all samples prior to the test were analysed using X-ray diffraction (XRD) (D8-ADVANCE) with monochromatic Cu Kα radiation (λ = 0.15418 nm) at room temperature. The surface morphology and chemical compositions of selected areas were investigated by using a scanning electron microscope (SEM) (FEI Quanta 600) equipped with an energy dispersive X-ray spectrometer (EDS) (EDAX Inc., Pleasanton, CA, USA).

### 2.2. Thermodynamic Model and Calculation Algorithm

During model construction, the Gibbs energy functions of the three elements V, Ti, and Fe in the present work are excerpts from Dinsdale’s SGTE (Scientific Group Thermodata Europe, Saint-Martin-d’Hères, France) compilation [22]. After our preliminary calculations, there are a total of three different types of phases present during their solidification process, which are the liquid phase, solution phases (bcc, fcc and hcp), and intermetallic compound phase (TiFe, TiFe_2_, and sigma). Thus, models used for all phases were established. For the liquid phase, its Gibbs energy is obtained by a substitutional solution model, which can be described by the following Redlich–Kister polynomial [23]:

(1) Gφ0=∑i=14xi0GiL+RT∑i=14xilnxi+∑i=13∑j=i+14xixjLi,j+∑i=12∑j=i+13∑k=j+14xixjxkLi,j,k

where *G_i_* and *x_i_* represent the Gibbs energy and mole fraction of element *i*, respectively. *T* and *R* represent the temperature and gas constant, respectively. *L* represents the interaction parameter among different elements.

The solution phases can be described by the following equation:

(2) GmϕT=xFeGFeϕT+xTiGTiϕT+xVGVϕT+RTxFelnxFe+xTilnxTi+xVlnxV+GmϕE

where *x_i_* represent the mole fractions and subscript *i* represents pure elements *V*, *Ti*, or *Fe*, respectively. The rightmost expression is the excess Gibbs energy, which can be calculated using the following Redlich–Kister equation:

(3) GmϕE=xFexTi∑jjLFe,TiϕxFe−xTij+xTixV∑jjLTi,VϕxTi−xVj+xFexV∑jjLFe,VϕxFe−xVj+xFexTixVLFe,Ti,Vϕ

where *L_i_,_j_* represents the interaction parameter among different elements, i.e., *V*, *Ti*, or *Fe*. Additionally, the contribution of magnetism to the Gibbs free energy of the bcc phase is considered in order to calculate accurately, and a relevant expression can be found in Refs [24,25] proposed by Inden et al. and Hillert et al.

The intermetallic compounds, Fe_2_Ti, FeTi, and sigma were treated as (Fe,Ti,V)_2_(Fe,Ti,V), (Fe,Ti,V)_0.5_(Fe,Ti,V)_0.5_, and (Fe,Ti,V)_10_(Fe,Ti,V)_20_, respectively, according their crystal structure and solubility, which have been previously reported by Guo, Hillert, and Sundman et al. [26,27].

In addition, the solidification path of different alloys was calculated by Thermo-Calc software (TCW 3) coupled with our previously developed micro-segregation model [28,29]. All thermodynamic parameters are obtained from the Thermo-Calc software and participate in each step of the calculation. In the calculation of the primary phase and binary eutectic solidification, phase volume fraction (*fs*) is selected as the control variable, while temperature (*T*) is used as the control parameter for ternary invariant reaction. Other details can be found in our previous work [30]. Table 3 summarises the optimised thermodynamic parameters and related physical parameters.

## 3. Results and Discussion

Figure 2 shows the calculated results (blue line) of the V-Ti-Fe ternary phase diagram. Clearly, a quasi-peritectic equilibrium, L + TiFe_2_ → bcc-(V, Ti) + TiFe (1139.2 K) exists in the calculated liquidus projection. Furthermore, there are three phase regions, i.e., bcc-(V, Ti), TiFe, and TiFe_2_, around this ternary invariant reaction. Of these regions, the area is minimal for TiFe, moderate for TiFe_2_, and maximal for bcc-(V, Ti). According to the V-Ti binary phase diagram [31], the infinite solid solution between Ti and V elements may be one of the reasons for the larger bcc-(V, Ti) region. These equilibrium calculations are basically consistent with results reported by Guo et al. [16], although some differences exist in the boundaries of the TiFe_2_ region. However, there is a discrepancy between our calculation results and those (see pink dot dash) of Massicot et al. [17]. Typically, our calculations show that quasi-peritectic equilibrium occurs at the V_13.63_Ti_55.16_Fe_31.21_ composition, which is significantly different from the V_18.56_Ti_49.03_Fe_32.41_ reported by Massicot et al., see Table 4 and Figure 2. In addition, the TiFe region reported by Massicot et al. is much larger and even includes the ternary quasi-peritectic equilibrium points calculated in the present work. The reasons for this discrepancy can be attributed the different thermodynamic model, database, as well as relevant parameters used in this work and the literature.

**Figure 3:**
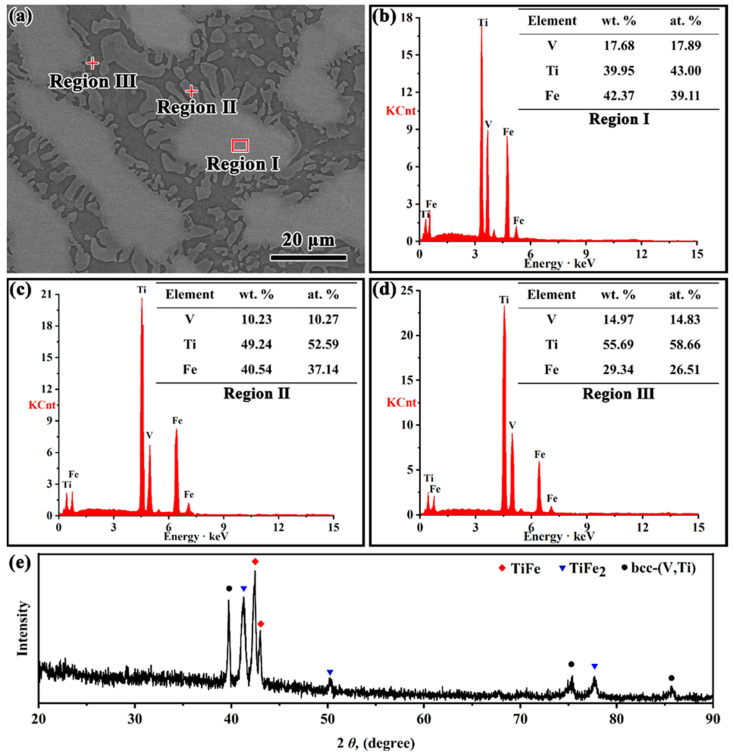
EDS and XRD results of as-cast V_15_Ti_50_Fe_35_ (#7) alloys. (**a**) SEM micrograph, (**b**–**d**) are the element distribution results for region I, II and III, respectively, (**e**) XRD patterns.

**Figure 4:**
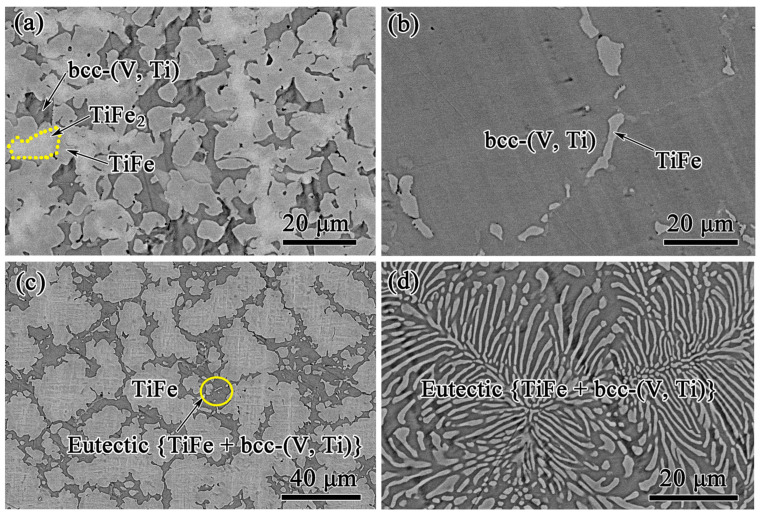
FE-SEM surface images of as-cast V-Ti-Fe alloys. (**a**) #8 (V_12.5_Ti_50_Fe_37.5_), (**b**) #9 (V_20_Ti_55_Fe_25_), (**c**) #10 (V_5_Ti_55_Fe_40_), and (**d**) #11 (V_5_Ti_65_Fe_30_).

To verify the accuracy of the calculated phase equilibrium in this work, 5 V-Ti-Fe alloys (marked as #7–#11 in Figure 2) were selected in different phase regions, and their crystal structures, microstructures, and solidification characteristics were studied, as shown in Figure 3 and Figure 4. Alloy #7 (V_15_Ti_50_Fe_35_) is composed of three different phases, which are TiFe_2_, TiFe, and bcc-(V, Ti), respectively, see Figure 3a,e. The phase marked as region I contains the most Fe content (39.11 at.%) and is therefore identified as TiFe_2_, Figure 3b. This phase, as a primary phase, preferentially solidifies in the liquid alloy. Using a similar elemental analysis method, the phases of regions II and III are identified as TiFe and bcc-(V, Ti), respectively, as shown in Figure 3c,d. These two phases form a coarse eutectic structure that surrounds the primary TiFe_2_ phase. Similar structural features can be found in the V_12.5_Ti_50_Fe_37.5_ (#8) alloy, although the amount of primary TiFe_2_ phases is relatively low, ca. 12%, see Figure 4a. These results suggest that these two alloys first undergo primary TiFe_2_ phase solidification, followed by ternary quasi-peritectic transformation, L + TiFe_2_ → bcc-(V, Ti) + TiFe, resulting in eutectic {bcc-(V, Ti) + TiFe} structure, see our results (blue line) in Figure 2.

Unlike the structures in alloys #7 and #8, the primary phases in V_17.5_Ti_50_Fe_32.5_ (9#) and V_22.5_Ti_52.5_Fe_25_ (10#) alloys are bcc-(V, Ti) and TiFe, respectively, with a small amount of eutectic distribution at the edges, see Figure 4b,c. Obviously, these two alloys undergo the following reactions in sequence: [L → bcc-(V, Ti) or TiFe] and [L → bcc-(V, Ti) + TiFe]. In comparison, alloy #11 (V_20_Ti_55_Fe_25_) only undergoes eutectic reaction of L → bcc-(V, Ti) + TiFe, resulting in a fully eutectic structure, Figure 4d. This is mainly attributed to its unique position on the eutectic univariant line, as shown in Figure 2. 

All the above experimental data are consistent with the calculated phase diagram in this work but differ significantly from previous results reported by Massicot et al. [17]. Specifically, according to the calculation results of Massicot et al., the primary phase of alloys #7 and #8 should be TiFe rather than TiFe_2_, see Figure 2. However, the opposite result was observed in our experiment, where the primary phase was the TiFe_2_ phase, which was confirmed by subsequent SEM characterisation in Figure 3 and Figure 4a. Similar structural characteristics also exist in the (Ti_70.5_Fe_29.5_)_100−x_V_x_ alloys (x = 3, 5 and 7) reported by Han et al. [32], which contradict the calculation results of Massicot et al. In addition, the information related to the isothermal sections at 800 K and 1200 K reported by Guo and Wang et al. [16,33] is consistent with our calculated results, but differs from Massicot et al. The above analysis clearly indicates that compared to the previous studies, the phase equilibrium of the V-Ti-Fe system calculated in this work is dependable and more accurate. 

In addition, the calculation results also imply that dual-phase (bcc-(V, Ti) + TiFe) alloys used for hydrogen permeation are not located in the position of the quasi-binary V-Ti-Fe phase diagram, which is different from the recently reported Nb-Ti-Ni [34] or Nb-Ti-Co [35] system. This is mainly due to the movement of the quasi-peritectic equilibrium point, U1, towards the upper left corner, i.e., rich Ti angle. In this case, the TiFe_2_ phase zone will expand, thus narrowing the selection range of the dual-phase alloy composition. Otherwise, it is possible to form a TiFe_2_ phase, such as the previously studied V_15_Ti_50_Fe_35_ (#7) or V_12.5_Ti_50_Fe_37.5_ (#8) alloys, see Figure 3 and Figure 4a. Therefore, selecting compositions based on the calculated phase diagram above is worthwhile and challenging to study. To this end, a (Ti_65_Fe_35_)_100−x_V_x_ (x = 0…25) alloy series (marked as #1–#6 in Figure 2) was designed. Prior to the solidification experiment, a simulation calculation study of the solidification path was conducted for these alloys.

**Figure 5:**
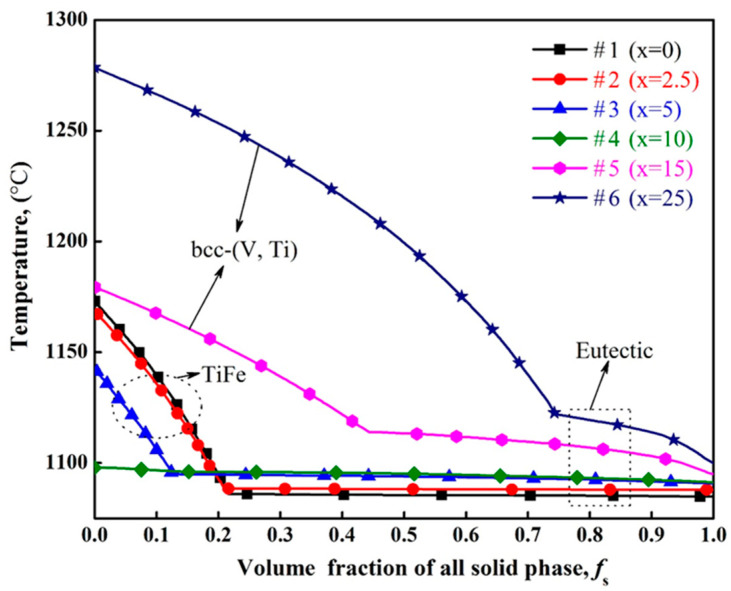
Solidification paths of as-cast V_x_(Ti_65_Fe_35_)_1−x_ alloys with #1 (Ti_65_Fe_35_), #2 (V_2.5_(Ti_65_Fe_35_)_97.5_), #3 (V_5_(Ti_65_Fe_35_)_95_), #4 (V_10_(Ti_65_Fe_35_)_90_), #5 (V_15_(Ti_65_Fe_35_)_85_), and #6 (V_20_(Ti_65_Fe_35_)_80_).

Figure 5 shows the calculated results of solidification paths for as-cast (Ti_65_Fe_35_)_100−x_V_x_ alloys. In alloys #1–#3, primary TiFe phase solidification, i.e., L → TiFe, occurs first, followed by binary eutectic solidification. Conversely, in #5 and #6 alloys, the primary phase is bcc-(V, Ti), not TiFe. As a special case, alloy #4 only undergoes a binary eutectic reaction, composed of the fully eutectic structure. It is worth noting that when the x value is equal to 0 at.%, the alloy composition is Ti_65_Fe_35_, and its eutectic structure is composed of TiFe and β-Ti phases. This is different from eutectic (TiFe and bcc-(V, Ti)) in other alloys. These results suggest that these investigated alloys can be divided into three types, namely hypoeutectic alloys (#1–#3) with primary TiFe, eutectic alloy (#4), and hypereutectic alloys (#5 and #6) with primary bcc-(V, Ti). Except for alloy #1, all alloys are composed of TiFe and bcc-(V, Ti) phases. These simulation results provide direction for the subsequent development of hydrogen permeation alloys, but further experimental verification is still needed.

**Figure 6:**
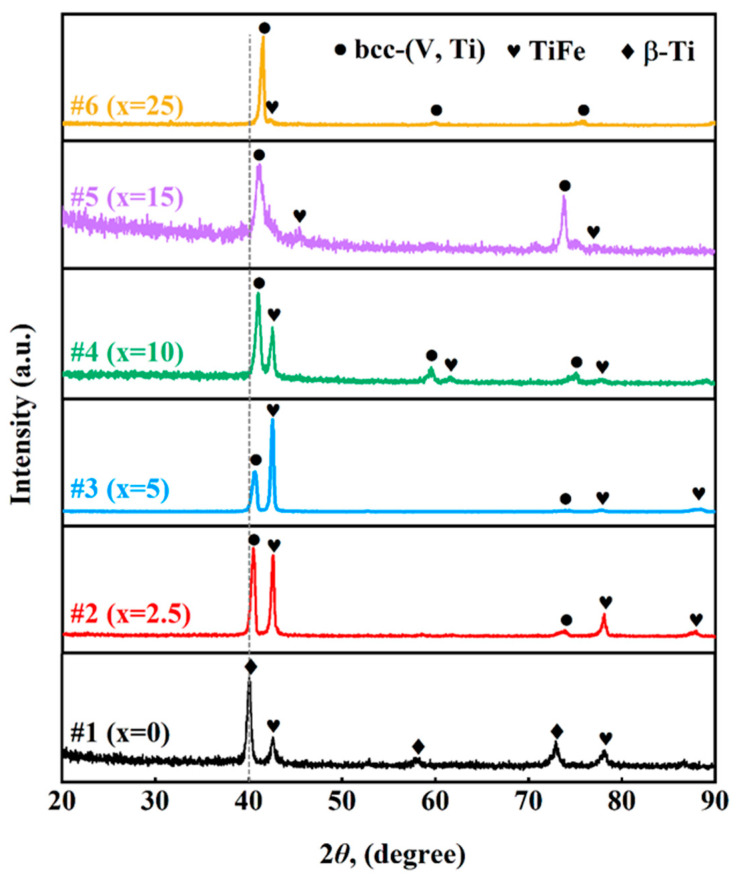
XRD diffractograms of the as-cast V_x_(Ti_65_Fe_35_)_1−x_ alloys with #1 (Ti_65_Fe_35_), #2 (V_2.5_(Ti_65_Fe_35_)_97.5_), #3 (V_5_(Ti_65_Fe_35_)_95_), #4 (V_10_(Ti_65_Fe_35_)_90_), #5 (V_15_(Ti_65_Fe_35_)_85_), and #6 (V_20_(Ti_65_Fe_35_)_80_).

**Figure 7:**
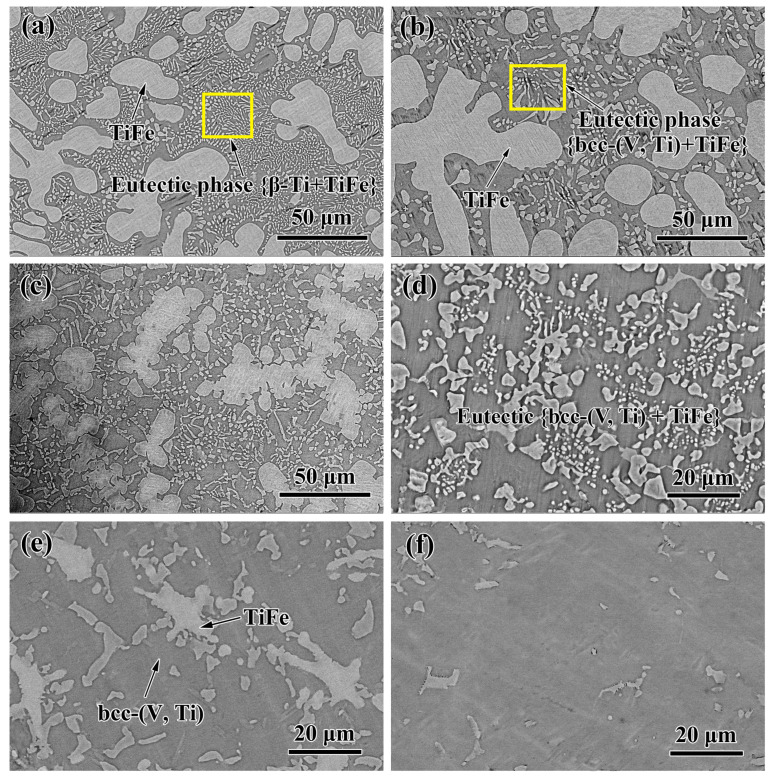
Typical BSE images of the as-cast V_x_(Ti_65_Fe_35_)_1−x_ alloys with (**a**) #1 (x = 0, Ti_65_Fe_35_), (**b**) #2 (x = 2.5, V_2.5_(Ti_65_Fe_35_)_97.5_), (**c**) #3 (x = 5, V_5_(Ti_65_Fe_35_)_95_), (**d**) #4 (x = 10, V_10_(Ti_65_Fe_35_)_90_), (**e**) #5 (x = 15, V_15_(Ti_65_Fe_35_)_85_), and (**f**) #6 (x = 25, V_25_(Ti_65_Fe_35_)_75_).

Accordingly, XRD and SEM characterisation was performed on all the alloys mentioned above, and the results are shown in Figure 6 and Figure 7. Clearly, alloy #1 (x = 0) consists of only TiFe and β-Ti phases, while other alloys, #2–#7, are mainly composed of TiFe and bcc-(V, Ti) phases, Figure 6. As the content of V increases (i.e., x↑), the main diffraction peak positions of the bcc-(V, Ti) solid solution phase gradually move to the right. According to the V-Ti phase diagram [31], the V phase can dissolve infinite elemental Ti even at temperatures below 573 K according to Vegard’s law, which means that an increase in V content in the bcc-(V, Ti) phase leads to a decrease in lattice parameter. In fact, the atomic radius of V (0.132 nm) is smaller than that of Ti (0.146 nm), so lattice shrinkage will inevitably occur after replacing Ti with V.

By further analysis of the BSE microstructures of each sample in Figure 7, hypoeutectic structure is composed of primary TiFe dendrites and an interdendritic eutectic can be found in alloys #1–#3; the primary phase gradually decreases with the increase in V content, Figure 7a–c. Compared to bcc-(V, Ti), the eutectic containing β-Ti is significantly finer. The reason for this phenomenon is currently unknown, perhaps related to the solidification characteristics of these two phases. For alloy #4, it is completely composed of divorced eutectic (TiFe + bcc-(V, Ti)), Figure 7d. In contrast, as the V content further increases (#5 → #6), the eutectic structure gradually decreases, but the primary bcc-(V, Ti) phase increases, as shown in Figure 7e,f.

Combining all results in Figure 5, Figure 6 and Figure 7, it can be confirmed that there is a dual-phase alloy composition in the V-Ti-Fe system, such as the (Ti_65_Fe_35_)_100−x_V_x_ (x = 0…25) alloy series studied here. Nonetheless, the dual phases are not located in the V-Ti-Fe pseudo-binary isopleth, but rather near the Ti-rich angle. In addition, the above experimental results are consistent with the simulation results in Figure 5, further demonstrating the accuracy of the calculated phase diagram in this work. In short, the discovery of these dual-phase alloys provides a reference for subsequent hydrogen permeation material design. Subsequently, the hydrogen permeation performance of these alloys was measured. 

**Figure 8:**
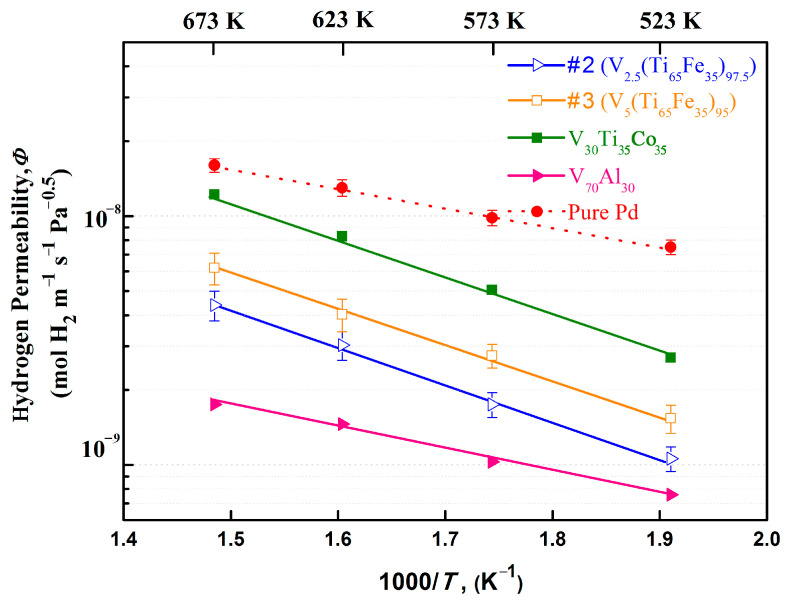
Temperature dependence of hydrogen permeability for V_x_(Ti_65_Fe_35_)_1−x_ alloys as well as V_30_Ti_35_Co_35_ [36], V_70_Al_30_ [37], and pure Pd [3,38].

In the hydrogen permeation test, it was not possible to measure the performance of alloys #4–#6 due to their hydrogen embrittlement. Furthermore, the H_2_ flux of the alloy #1 was too low, being smaller than the measurement range of the hydrogen permeation instrument, so its hydrogen permeation performance could not be obtained. In this case, only the hydrogen permeability of alloys #2 and #3 were successfully measured, and their results are shown in Figure 8. For comparison, homologous alloys such as V_30_Ti_35_Co_35_ [36], V_70_Al_30_ [37], and pure Pd [3,38] are also listed in the figure. Clearly, for any membrane, the two parameters of temperature and hydrogen permeability satisfy a linear relationship. This implies that the hydrogen transport through the membrane follows Fick’s first law and is diffusion-limited rather than surface-adsorption-process-limited. At each measurement temperature, the permeability value of #3 alloy was superior to that of #2, which indicates that the eutectic, especially the inner bcc-(V, Ti) phase, is beneficial for hydrogen transport. Notably, the permeability of these two membranes was ca. 4.2–6.1 × 10^−9^ mol H_2_ m^−1^s^−1^Pa^−0.5^ at 673K, which is significantly lower than that (1.1–1.6 × 10^−8^ mol H_2_ m^−1^s^−1^Pa^−0.5^) of V_30_Ti_35_Co_35_ [36] and pure Pd [38]. Nevertheless, this is much higher than V_70_Al_30_ previously reported by Nishimura et al. [37].

**Figure 9:**
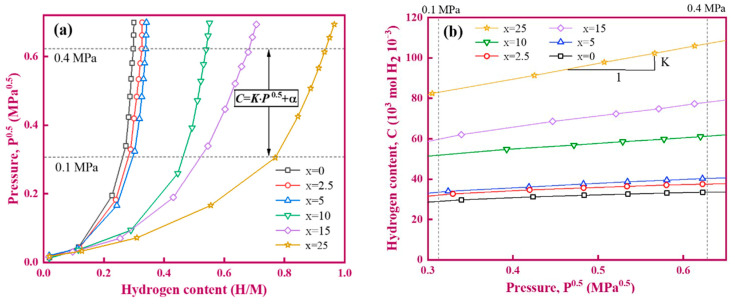
PCT curves of (Ti_65_Fe_35_)_1−x_V_x_ samples at 400 °C. (**a**) H/M vs P^0.5^; (**b**) C vs P^0.5^.

To further analyse the reasons for the above changes in permeability, the PCT curves of the (Ti_65_Fe_35_)_100−x_V_x_ alloys (#1–#6) were measured at 673 K, and the results are shown in Figure 9. For each alloy, as the pressure gradually increases, the hydrogen solubility continuously increases, Figure 9a. When the hydrogen pressure is less than 0.01 MPa, there is a linear relationship between the pressure and hydrogen concentration (C), and the fitting curve crosses the origin, indicating that their relationship conforms to Sievert’s law, as follows:

(4) $$C=K\cdot P^{0.5}$$

On the contrary, when pressure is within the range of 0.1–0.4 MPa, the PCT curve does not comply with Sievert’s law. After linear fitting, their relationship can be expressed as follows:

(5) $$C=K\cdot P^{0.5}+\alpha$$

According to equation (5), the hydrogen solubility K of alloys #1–#6 is obtained. In addition, the hydrogen diffusivity (D) can be further derived by using equation Φ = D × K. These parameters are summarised in Table 5. Obviously, hydrogen concentration is closely related to hydrogen pressure at a fixed V content. When the hydrogen pressure is below 0.4 MPa, the higher the hydrogen pressure, the higher the hydrogen concentration. Similarly, Kundalwal et al. [39] also demonstrated that the increment in hydrogen pressure favours the adsorption energies of molecular hydrogen by using molecular dynamic simulations. As the V content increases, the hydrogen solubility sharply increases, but the hydrogen diffusivity gradually decreases. This indicates that the hydrogen solubility is greater than the hydrogen diffusion in terms of its impact on hydrogen permeation performance. The two possible causes responsible for this phenomenon are microstructure changes and an increase in Ti content in bcc-(V, Ti) solid solution. On the one hand, compared to the compound TiFe phase, the solid solution bcc-(V, Ti) phase is more prone to hydrogen absorption [4,5,6,7,8,9,40]. In this case, alloys #4–#6, with more primary bcc-(V, Ti) phase, have relatively high hydrogen solubility and are more prone to hydrogen embrittlement compared to alloys #1 and #2 with more primary TiFe phase. For alloy #3, its microstructure is entirely composed of eutectic phases with more phase boundary. According to the results of Wu and Zhang et al. [41,42], these phase boundaries can act as a hydrogen trap, making hydrogen diffusion more tortuous and difficult. From this perspective, the increase in eutectic interface is also one of the reasons for the increase in K value.

On the other hand, as the V content increases, the bcc-(V, Ti) phase increases and the internal solid solution Ti atoms increase, see Table 1. Excessive Ti atoms will result in a higher hydrogen concentration in the alloys due to its lower enthalpy of hydride formation [46,47]. This will make the membrane more susceptible to hydrogen embrittlement. Overall, dual-phase V-Ti-Fe alloys with a V content below 10 at.%, especially V_5_(Ti_65_Fe_35_)_95_ (#3) alloy, have advantages in hydrogen permeation and hydrogen embrittlement resistance, which are worthy of further research. Next, the V_5_(Ti_65_Fe_35_)_95_ tubular alloy membrane was successfully prepared, and its microstructure and hydrogen permeation performance were studied.

**Figure 10:**
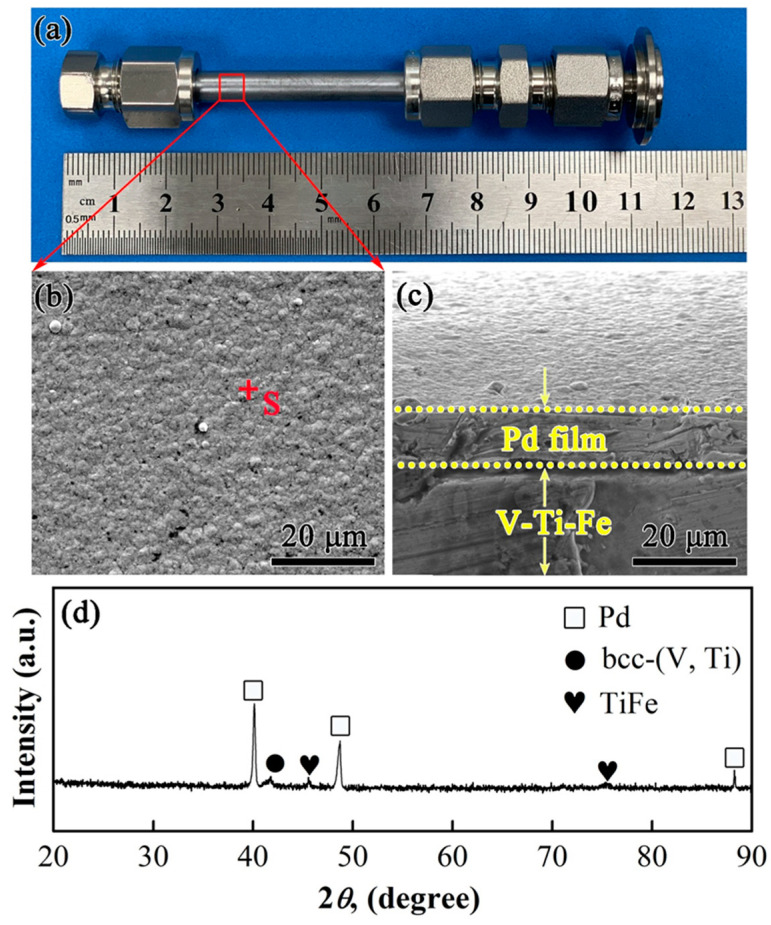
Microstructure characteristics of tubular V_5_(Ti_65_Fe_35_)_95_ sample prior to testing. (**a**) Appearance of tubular membrane with the left end capped, (**b**) SEM micrograph of Pd layer on the substrate surface, (**c**) cross-section of Pd catalyst layer on V_5_(Ti_65_Fe_35_)_95_ substrate, and (**d**) XRD pattern of tubular Pd-coated V_5_(Ti_65_Fe_35_)_95_ sample.

Figure 10 shows the appearance of the tubular V_5_(Ti_65_Fe_35_)_95_ membrane of 60 mm in length. After chemical plating with Pd, the film was a brightly coloured and a smooth surface without obvious defects such as pinholes and cracks, Figure 10a. Pd particles form a dense catalytic layer and grow according to the Stranski–Krastanov model [48], Figure 10b,c. The final thickness of the Pd film was about 10 μm. According to EDS testing results, the Pd content in the film was about 99.99 at.%, and it is evenly distributed on the surface of the substrate. The Pd diffraction peaks in the XRD pattern further confirm that the Pd film was successfully deposited on the substrate surface.

**Figure 11:**
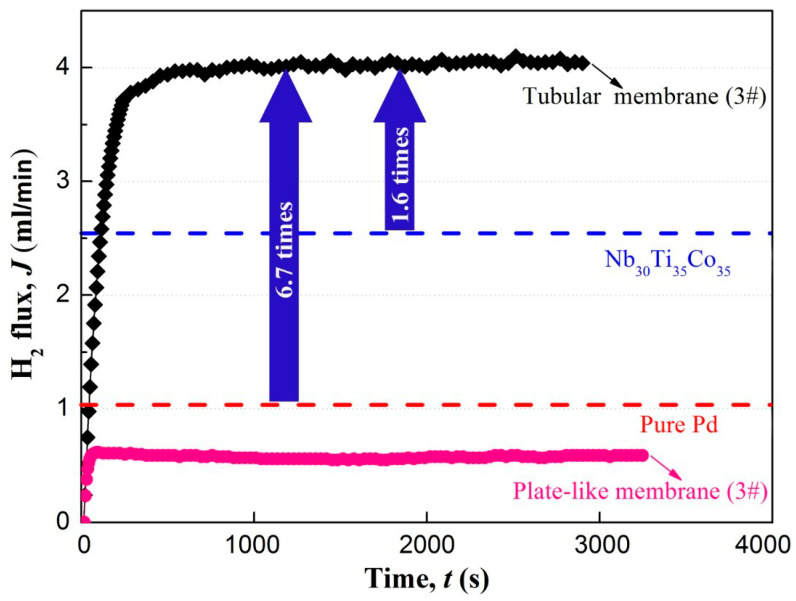
H_2_ flux with varying time for tubular and sheet V_5_(Ti_65_Fe_35_)_95_ samples. The blue and red dashed lines represent the test results of Nb_30_Ti_35_Co_35_ [36] and pure Pd [38], respectively.

Figure 11 shows the hydrogen permeation performance of the tubular V_5_(Ti_65_Fe_35_)_95_ membrane. When pressurised on the upstream side of the membrane, the H_2_ flux through the membrane gradually increased. After ~3 min, the hydrogen flow rate stabilised, and the final H_2_ flux was ca. 4.05 mL min^−1^, which is 6.7 times higher than that (0.6 mL min^−1^) of the counterpart, i.e., the plate-like V_5_(Ti_65_Fe_35_)_95_ membrane. In addition, the H_2_ flux of this tubular membrane is also higher than that of traditional Nb_30_Ti_35_Co_35_ or Pd sheet-like membrane [36,38] with a diameter of 12 mm under the same test conditions.

Finally, we summarised the advantages of tubular alloy membranes compared to previously developed plate-like ones in the field [49,50,51,52,53,54]. Generally, the total H_2_ flux (J) permeation through the membrane can be described as follows [55]:

(6) $$J=J’\times S=\frac{\phi \times \Delta p^{1/2}}{L}\times S$$

where *J*′ represent the H_2_ flux per unit area and is mainly influenced by the parameters of hydrogen permeability (*Φ*), pressure difference (∆*P*^1/2^), and membrane thickness (*L*). *S* is the effective hydrogen permeation area. Equation (6) indicates that there are at least two ways to increase the *J* value, namely choosing metals with high *J*’ and increasing *S*. Regarding the former, most research groups, such as Dolan, Hara, and Aoki et al. [9,15,46,56], mainly focused on developing a multicomponent alloy system with a high *Φ* value, or using special preparation techniques (i.e., melt-spinning and rolling [57]) to fabricate films with the least thickness, thereby increasing the *J*′ value during the hydrogen permeation process. Compared with rolling, melt-spinning offers significant advantages. This is mainly because it is not only a near-net shaping technology, but also can continuously produce membranes with minimum thickness. For example, we have successfully developed Nb_30_Ti_35_Co_35_ alloy ribbon (~25-mm wide) by this method [58]. After proper annealing, it shows a high hydrogen permeation flux and excellent durability. In addition, research on the use of directional solidification to prepare the hydrogen-permeable membranes has also been reported [59]. This technology can change the shape and distribution of phases, thus improving the hydrogen diffusivity and permeability. For example, the hydrogen permeability of Nb_19_Ti_40_Ni_41_ membrane [60] fabricated by using this technology is about two times than that of its as-cast counterpart. Although good results have been achieved in the early stage, the selection of higher performance alloy components is relatively difficult due to the lack of phase diagrams in relevant ternary or quaternary alloys. For example, in the early research of Nb-Ti-Ni [8] or Ta-Ti-Ni [61] systems, Ishikawa et al. [61] needed to try various components to obtain satisfactory alloys with maximum hydrogen permeability. In addition, for membranes prepared using special preparation techniques, multiple work procedures are required to achieve the desired thickness. These additional steps will undoubtedly increase manufacturing costs.

**Figure 12:**
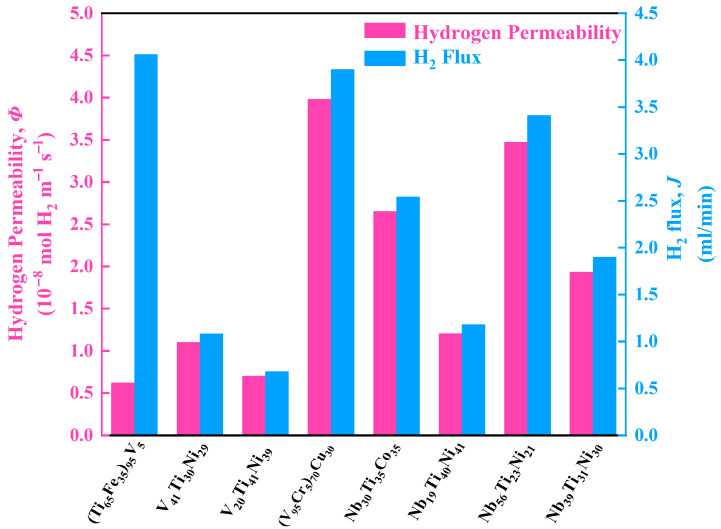
Comparison of hydrogen permeability and H_2_ flux of representative dense Nb- and V-based alloy membranes [49,50,51,52,53,54] for gaseous hydrogen separation.

In contrast, the tubular membrane studied in this work eliminates the complex preparation process and can be produced using traditional electric arc furnace casting. This kind of method can reduce costs and make the tubular membrane more adaptable. More importantly, the extension of the length of the tubular membrane can overcome the problem of smaller H_2_ flux (*J*) caused by low hydrogen permeability. For example, although the permeability value of V_5_(Ti_65_Fe_35_)_95_ is much lower, the H_2_ flux of tubular V_5_(Ti_65_Fe_35_)_95_ membrane is higher than that of other V- or Nb-based plate-like films, see Figure 12. Typically, the hydrogen permeability of V_5_(Ti_65_Fe_35_)_95_ is 6.15 × 10^−9^ mol H_2_ m^−1^s^−1^Pa^−0.5^ at 400 °C, which is about (1/2~1/5) times that of V- or Nb-based membranes and 1/3 times that of the commercial Pd-based (Pd_75_Ag_25_ [43], Pd_87_Au_13_ [44], and Pd_60_Cu_40_ [45]) alloys. Despite all this, the tubular V_5_(Ti_65_Fe_35_)_95_ membrane shows a significant increase in total H_2_ flux. This advantage will be more evident in the high permeability alloy series such as recently developed Nb-Hf-Co [30] and Nb-Ti-Fe-Cu [62] systems. In practical applications, due to volume limitations, if the length of a single pipeline is limited, 10 or more pipelines can be installed in the hydrogen permeation chamber to meet practical needs. This is difficult to achieve for sheet-like membranes.

In short, this work not only established the phase equilibrium parameters of the V-Ti-Co system, but also successfully developed tubular V_5_(Ti_65_Fe_35_)_95_ membranes. This provides important reference and theoretical basis for the development of subsequent functional materials using the phase diagram of this system in the future.

## 4. Conclusions

In this study, the phase equilibria of the V-Ti-Fe system were reconstructed by means of CALPHAD method. One ternary quasi-peritectic reaction was confirmed in this system, and the calculated composition of this four-phase invariant reaction is 13.63 at.% V, 55.16 at.% Ti, and 31.21 at.% Fe. The largest solubilities of V in TiFe and TiFe_2_ phases are about 13.63 at.% and 38.11 at.%, respectively. It can accurately and reasonably predict the microstructure characteristics and solidification parameters of new alloy series (Ti_65_Fe_35_)_100−x_V_x_. When the x value is equal to 10, the alloy is composed of fully eutectic phases. Above or below this value, there will be additional formation of primary bcc-(V, Ti) and TiFe phases in the structure. Of these alloys, (Ti_65_Fe_35_)_97.5_V_2.5_ and (Ti_65_Fe_35_)_95_V_5_ has been proven to exhibit relatively high hydrogen permeability. Finally, a 600 mm-long tubular (Ti_65_Fe_35_)_95_V_5_ membrane for hydrogen permeation was prepared successfully and showed a very high H_2_ flux of 4.06 mL min^−1^, which is ca. 6.7 times higher than the plate-like counterpart (0.61 mL min^−1^) under the same test conditions.

## Figures and Tables

**Table 1:** The compositions, constituting phases, as well as the chemical composition of V-Ti-Fe alloys investigated in the present work.

No.	Samples	Measured Alloy Compositions (at.%)	Constituting Phases	Chemical Composition of Primary bcc-(V, Ti)		
				V	Ti	Fe
#1	Ti_65_Fe_35_	Ti_64.9_Fe_35.1_	TiFe, eutectic {*β*-Ti + TiFe}	—	—	—
#2	V_2.5_(Ti_65_Fe_35_)_97.5_	V_2.4_Ti_63.4_Fe_34.2_	TiFe, eutectic {bcc-(V, Ti) + TiFe}	—	—	—
#3	V_5_(Ti_65_Fe_35_)_95_	V_5.1_Ti_61.9_Fe_33_	TiFe, eutectic {bcc-(V, Ti) + TiFe}	—	—	—
#4	V_10_(Ti_65_Fe_35_)_90_	V_9.9_Ti_58.5_Fe_31.6_	Eutectic {bcc-(V, Ti) + TiFe}	—	—	—
#5	V_15_(Ti_65_Fe_35_)_85_	V_15.1_Ti_55.3_Fe_29.6_	bcc-(V, Ti), eutectic {bcc-(V, Ti) + TiFe}	10.27	52.59	37.14
#6	V_25_(Ti_65_Fe_35_)_75_	V_24.8_Ti_48.8_Fe_26.4_	bcc-(V, Ti), eutectic {bcc-(V, Ti) + TiFe}	16.10	56.82	27.08
#7	V_15_Ti_50_Fe_35_	V_15.1_Ti_49.5_Fe_35.4_	TiFe_2_, eutectic {bcc-(V, Ti) + TiFe}	—	—	—
#8	V_12.5_Ti_50_Fe_37.5_	V_12.4_Ti_49.8_Fe_37.3_	TiFe_2_, eutectic {bcc-(V, Ti) + TiFe}	—	—	—
#9	V_17.5_Ti_50_Fe_32.5_	V_17.3_Ti_50.1_Fe_32.6_	bcc-(V, Ti), eutectic {bcc-(V, Ti) + TiFe}	11.14	54.96	33.90
#10	V_22.5_Ti_52.5_Fe_25_	V_22.4_Ti_52.4_Fe_25.2_	TiFe, eutectic {bcc-(V, Ti) + TiFe}	—	—	—
#11	V_20_Ti_55_Fe_25_	V_19.9_Ti_55.2_Fe_24.9_	Eutectic {bcc-(V, Ti) + TiFe}	—	—	—

**Table 2:** The chemical plating formula.

Materials	Content	Temperature (°C)
PdCl_2_	2 g/L	50 ± 1
HCl (36%)	10 mL/L	
NaH_2_PO_2_·H_2_O	10 g/L	
NH_4_Cl	27 g/L	
NH_3_·H_2_O (28%)	16 mL/L	
pH	9.8 ± 0.2	

**Table 3:** The thermodynamic parameters used in the present work.

Parameters		Values	Ref.
Solidification shrinkage		0.032	[15,23,27]
The distance of secondary dendrite (μm)		0.12	Calculated
V–Ti–Fe	$D_{Fe}^{\alpha }$ (mm^2^ s^−1^)	14 exp(−14,000/*T*)	[15,23,27]
	$D_{Ti}^{\alpha }$ (mm^2^ s^−1^)	22.3 exp(−11,000/*T*)	[15,23,27]
*L_bcc-V_* (J mol^−1^)		17,965 + 6.35*T* + (−6897 + 1.65*T*) × (*x*_V_ − *x*_Ti_)	Present work
*L*_TiFe_ (J mol^−1^)		−698+2.56*T* + 5687 × (*x*_Ti_ − *x*_Fe_)	Present work
*L*_Fe2Ti_ (J mol^−1^)		15,667+3.58*T* − 69.35 × (*x*_Ti_ − *x*_Fe_)	Present work
Solidification/cooling rates *R_f_* (s^−1^)		300	Calculated
Step length of α (Δ*f*_s_)		0.0025	Initial value
Step length of binary eutectic Δ*T* (°C)		0.25	Initial value
Specific heat (*S* and *L*) *c_PS_*, *c_PL_*(J kg^−1^K^−1^)		1263, 1789	[15,23,27]
Thermal conductivity (solid) *λ_S_* (W m^−1^K^−1^)		237	[15,23,27]
Thermal conductivity (liquid) *λ_L_* (W m^−1^K^−1^)		162	[15,23,27]
Liquidus temperature *T_liq_* (°C)		Depends on composition	By Thermo-Calc (Stockholm, Sweden)

**Table 4:** Invariant reactions with liquid phases of V-Ti-Fe system.

No.	Invariant Reaction	Temperature (°C)	Compositions of the Liquid Phases (at.%)			References
			*x* (V)	*x* (Ti)	*x* (Fe)	
U_1_	L+TiFe_2_→TiFe+bcc-(V, Ti)	1140	13.63	55.16	31.21	The present work
U_1_’	L+TiFe_2_→TiFe+bcc-(V, Ti)	1140	18.56	49.03	32.41	Massicot et al. [17]
U_1_”	L+TiFe_2_→TiFe+bcc-(V, Ti)	1140	11.94	56.51	31.55	Guo et al. [16]

**Table 5:** The values of hydrogen permeability, hydrogen solubility and hydrogen diffusivity for the V_x_(Ti_65_Fe_35_)_1−x_ alloys (#1…#6).

No.	Samples	Hydrogen Permeability	Hydrogen Solubility	Hydrogen Diffusivity
		[mol H_2_ m^−1^ s^−1^ Pa^−0.5^]	[mol H_2_ m^−3^ Pa^−0.5^]	[10^−9^m^2^ s^−1^]
#1	Ti_65_Fe_35_	—	13.48	—
#2	V_2.5_(Ti_65_Fe_35_)_97.5_	4.24 × 10^−9^	16.41	2.58
#3	V_5_(Ti_65_Fe_35_)_95_	6.15 × 10^−9^	21.03	2.92
#4	V_10_(Ti_65_Fe_35_)_90_	—	27.72	—
#5	V_15_(Ti_65_Fe_35_)_85_	—	55.62	—
#6	V_25_(Ti_65_Fe_35_)_75_	—	76.03	—
—	V_30_Ti_35_Co_35_ [36]	1.55 × 10^−8^	32.55	47.61
—	V_70_Al_30_ [37]	1.21× 10^−9^	20.16	0.6
—	Pd [3,38]	1.6 × 10^−8^	4.19	38.18
—	Pd_75_Ag_25_ [43]	1.48 × 10^−8^	—	—
—	Pd_87_Au_13_ [44]	1.12 × 10^−8^	—	—
—	Pd_60_Cu_40_ [45]	1.09 × 10^−8^	—	—

## Data Availability

The raw/processed data required to reproduce these findings cannot be shared at this time as the data also forms part of an ongoing study.
